# Supplementary material for: Rapid, simplified whole blood-based multiparameter assay to quantify and phenotype SARS-CoV-2 specific T cells
Source: Eur Respir J. Author manuscript; Available in PMC 2022 Jan 15. (PMC8215505; doi:10.1183/13993003.00285-2021)
Supplement: Supplementary Material [file EMS129596-supplement-Supplementary_Material.pdf]

Online supplement for:

Rapid, simplified whole blood-based multiparameter assay to quantify and phenotype SARS-CoV-2 specific T cells

Catherine Riou[1,2,*], Georgia Schäfer[1,3,4], Elsa du Bruyn[1,5], Rene T. Goliath[1], Cari Stek[1,5,6], Huihui Mou[7], Deli Hung[8], Katalin A. Wilkinson[1,5,9], Robert J. Wilkinson[1,5,6,9]

[1] Wellcome Centre for Infectious Disease Research in Africa and Institute of Infectious Disease and Molecular Medicine, University of Cape Town, Observatory 7925, South Africa
[2] Division of Immunology, Department of Pathology, University of Cape Town, Observatory 7925, South Africa
[3] International Centre for Genetic Engineering and Biotechnology (ICGEB) Cape Town, Observatory 7925, South Africa
[4] Division of Medical Biochemistry and Structural Biology, Department of Integrative Biomedical Sciences, University of Cape Town, Observatory 7925, South Africa
[5] Department of Medicine, University of Cape Town, Observatory 7925, South Africa
[6] Department of Infectious Diseases, Imperial College London, W12 0NN, UK
[7] Department of Immunology and Microbiology, The Scripps Research Institute, Jupiter, FL 33458, USA
[8] Department of Immunology and Microbiology, The Scripps Research Institute, La Jolla, CA 92037, USA
[9] The Francis Crick Institute, London, NW1 1AT, UK

Supp Table 1

Markers	Fluorochrome	Clone	Company	Cat. Number	Role
**CD3**	BV650	OKT3	BioLegend	317323	Lineage
**CD4**	BV785	OKT4	BioLegend	317428	
**CD8**	BV510	RPA-T8	BioLegend	301048	
**CD45RA**	Alexa 488	HI100	BioLegend	304114	Memory differentiation
**CD27**	PE-Cy5	1A4CD27	Beckman	6607107	
**CD38**	APC	HIT2	BD Bioscience	555462	Activation
**HLA-DR**	BV605	L243	BioLegend	307640	
**Ki67**	PerCP-Cy5.5	B56	BD Bioscience	561284	
**PD-1**	PE	EH12.2H7	BioLegend	329906	
**GrB**	BV421	GB11	BD Bioscience	563388	Cytotoxic potential
**IFNɣ**	BV711	4S.B3	BioLegend	502540	Functions
**TNFα**	PE-Cy7	MAB11	BioLegend	502930	
**IL-2**	PE/Dazzle™ 594	MQ1-17H12	BioLegend	500344	

Supp table 1: Description of the antibody panel used in the study.

Supp Figure 1

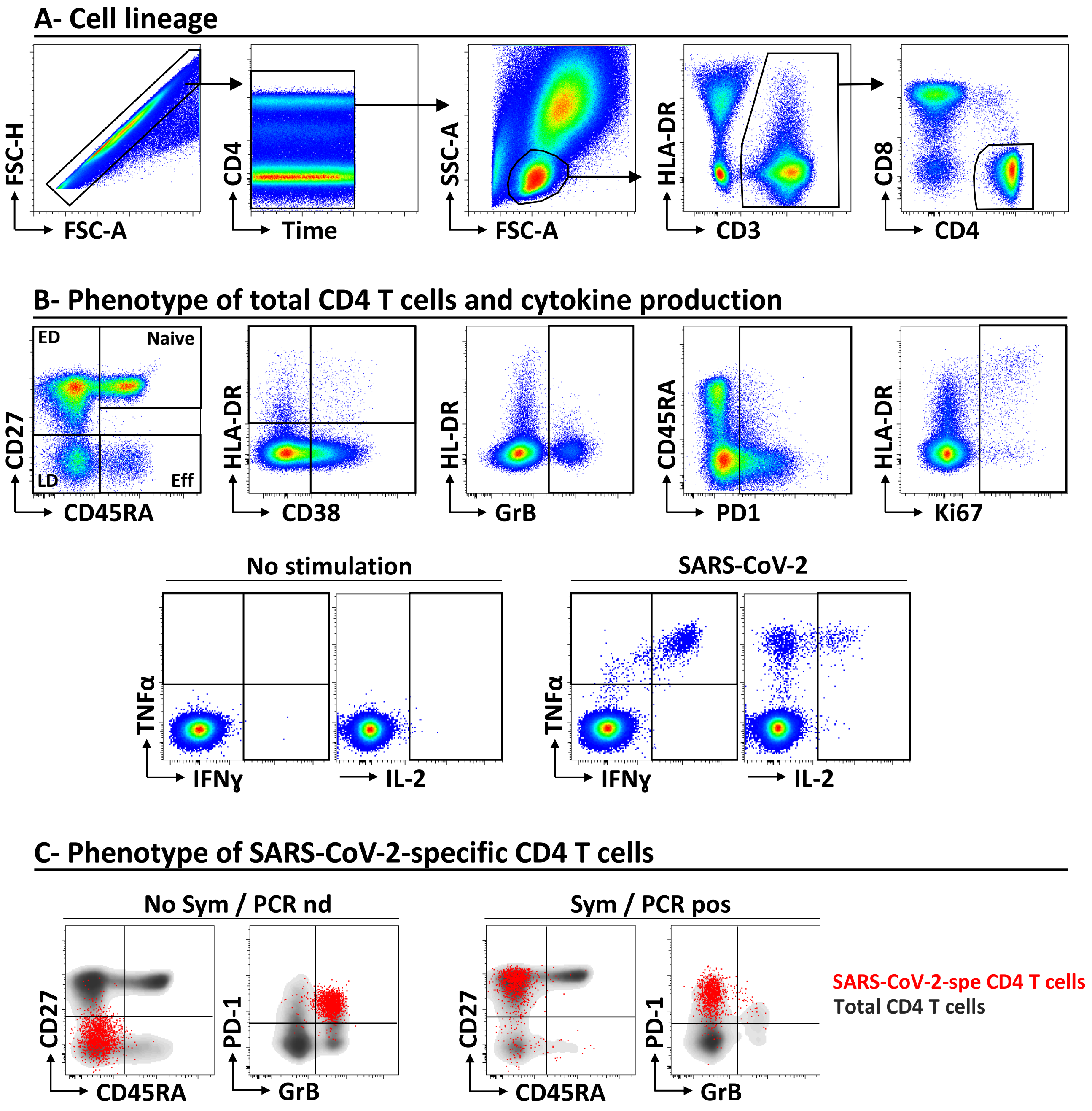

Supp Figure 1: Gating strategy used to identify SARS-CoV-2 specific CD4 T cells and defined their phenotypic characteristics. **A-** Cell lineage. **B-** Phenotyping of total CD4 T cell and cytokine production in response to SARS-CoV-2 peptides. **C-** Phenotype of SARS-CoV-2 responding CD4 T cells in one participant with no symptom (left) and one COVID-19 confirmed participant (right). SARS-CoV-2-specific CD4 T cells (expressing any measured cytokine) are depicted in red.

Supp Figure 2

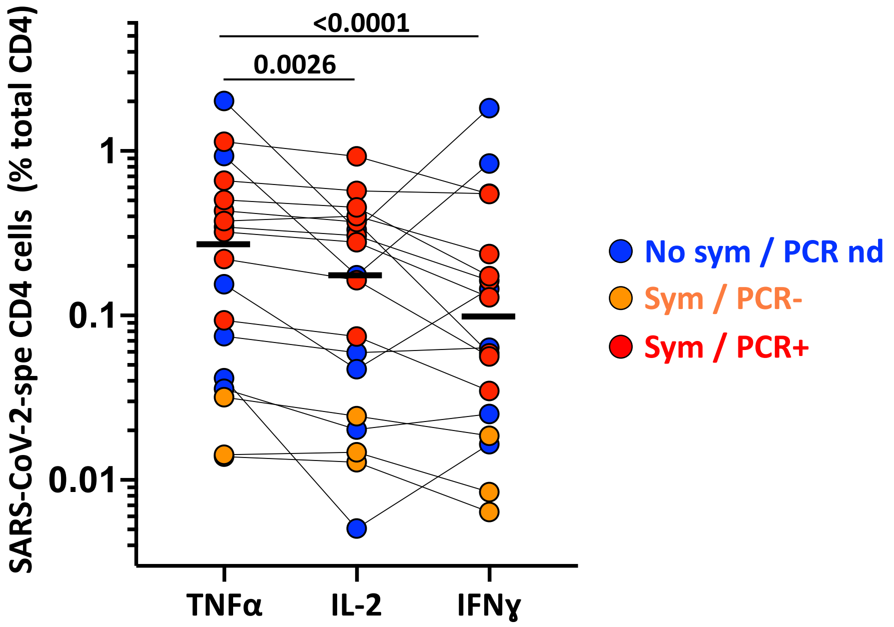

Supp Figure 2: Comparison of magnitude of SARS-CoV-2-specific CD4 T cell response expressing TNFα, IL-2 or IFNɣ in participants exhibiting a detectable response to SARS-CoV-2 peptide pool. Each dot represent a participant and is color-coded according to clinical characteristics (Blue represents no symptoms, no SARS-CoV-2 PCR performed; Orange: self-reported symptoms, SARS-CoV-2 PCR negative and Red: self-reported symptoms, SARS-CoV-2 PCR positive). Black bars indicate medians. Statistical comparisons were performed using a nonparametric paired Friedman test.
